# Outcome Comparison of Totally Implantable Venous Access Device Insertions Between Surgeons and Radiologists in Australia

**DOI:** 10.7759/cureus.23244

**Published:** 2022-03-17

**Authors:** Darius Dastouri, William T McSweeney, Matthew Leaning, Rasika Hendahewa

**Affiliations:** 1 General Surgery, Caboolture Hospital, Brisbane, AUS

**Keywords:** success, complications, interventional radiology, surgeons, tivad

## Abstract

Background

The need for chemotherapy treatment is increasing with the growing incidence of cancer worldwide. The insertion of totally implantable venous access devices (TIVADs) is commonly performed by surgeons and radiologists, but the procedures are not without complications. The primary outcome of this review outlines TIVAD insertion success and complication rates between general surgeons and radiologists. The secondary goal of this study is to help identify areas for improvement and consideration when performing TIVAD insertion.

Methodology

This was a descriptive, three-year, retrospective multicentre study of oncological patients who underwent TIVAD insertion by either general surgeons or radiologists at two peripheral Brisbane hospitals.

Results

Surgeons performed 61 percutaneous subclavian vein cannulations, 29 ultrasound-guided internal jugular veins, and seven open cephalic veins cut-down TIVAD insertions (n=97). Overall surgical success was 81.4%, with the internal jugular (89.7%) having the highest success rate followed by the open cut-down (85.7) and subclavian approaches (77.0%). The overall surgical complication rate was 16.4%, with five pneumothorax, five port malfunctions, three haemorrhages, two infections, one thrombus, and one mediastinal injury. Each pneumothorax was associated with subclavian cannulation attempts. Two haemorrhages were associated with both open cephalic and subclavian attempts. Radiologists performed 248 ultrasound-guided internal jugular vein TIVAD insertions (n=248) with 247 successful first attempts (99.5%). Within the radiology group, there was an overall complication rate of 15.3% with 22 infections, 14 port malfunctions, one haemorrhage, and 1 mediastinal injury.

Conclusion

Ultrasound-guided internal jugular vein TIVAD insertion had the highest first attempt success rate in both the surgical and radiology groups.

## Introduction

Central venous catheterization is used for patients who need long-term venous access for the infusion of chemotherapeutic agents [1]. Various methods of indwelling central venous access exist, including external catheterization (such as Hickman or Broviac catheters) and totally implantable venous access devices (TIVADs), commonly known as “port-a-caths” [2]. By incising the skin and implanting a port-a-cath underneath the subcutaneous tissue near its venous insertion site, TIVADs have been shown to have low complications rates with higher levels of reported patient quality of life [2-4]. As such, TIVAD insertion is becoming increasingly more common as the number of new cancer cases rises to approximately 14.1 million worldwide annually [5].

There are three main methods for inserting TIVADs: open venous cut-down of the cephalic vein, percutaneous subclavian cannulation, and ultrasound scan (USS)-guided cannulation of a central vein [2,5-6]. Although the open venous cut-down technique can be used on either the cephalic or the subclavian vein, the usual approach requires a surgeon trained in TIVAD insertion to dissect the deltopectoral groove and directly cannulate the cephalic vein [2]. The open venous cutdown has the added benefit of a “rescue technique” where a modified Seldinger technique is used to insert a guide-wire and vein dilator (with a peel-away sheath), yielding primary cannulation success rates of at least 90% [5,7]. In a percutaneous subclavian approach, the catheter is percutaneously advanced using the Seldinger technique after careful landmarking [2,6]. USS-guided cannulation of a central vein, such as the internal jugular or subclavian vein, is performed similarly to closed percutaneous cannulation but with the aid of ultrasound guidance followed by tunnelling of the port to the more accessible areas such as the upper chest [6]. Both percutaneous and USS-guided cannulation can be performed by either a surgeon or a radiologist and have been shown to have high rates of success on initial cannulation [2,5-6].

Complications of TIVAD insertion can be categorized as either early or late. Common early complications include haemothorax, pneumothorax, port-site haematoma, air embolism, cardiac arrhythmias, pericardial tamponade, and brachial plexus injuries [8-9]. Late complications include fibrin blockage, blood-borne infections, thrombosis, port site infection and inversion, superior vena cava erosion and perforation, and catheter dysfunction, rupture, and migration [10-11].

Current evidence suggests that there is no difference between TIVAD cannulation success and the open cut-down technique performed by surgeons compared to fluoroscopic-guided subclavian cannulation performed by radiologists [12]. This review is unique in that it is the first, to our knowledge, to directly compare all outcomes of general surgeons and radiologists who both commonly perform TIVAD insertions in peripheral and rural hospitals. The primary goal of this study is to compare the success rates and complication rates of TIVAD insertions between general surgeons and interventional radiologists. The secondary goal of this study is to help identify areas for improvement and consideration when performing TIVAD insertion.

## Materials and methods

A descriptive, three-year, multicentre, retrospective cohort review was performed between July 2017 and September 2020 at two peripheral hospitals in Brisbane, Australia. The study investigated adult oncological patients, 18 years and older, undergoing TIVAD insertion by general surgeons at Caboolture Hospital or radiologists at Redcliffe Hospital. The general surgical service at Caboolture Hospital employed six accredited Royal Australian College of Surgeons (RACS) surgeons who performed TIVAD insertions either surgically or under ultrasound guidance during this time period. Redcliffe Hospital radiologists are affiliated with the private company IMED Radiology, which employed seven consultant radiologists who routinely performed TIVAD insertions under ultrasound guidance during this period. Both hospitals only used low-profile BardPorts^TM^ (model: 0603870).

Caboolture Hospital protocol

All patients underwent a local multidisciplinary team meeting (MDT) to determine ongoing, appropriate management of their malignancy and the need for long-term venous access. Informed written consent was gained prior to conducting the procedure. The procedure was performed under general anaesthetic when patient performance status allowed. All procedures were performed as a day case in the operating theatre with both a surgical and an anaesthetic consultant present. All patients were prepped using alcoholic betadine or 2% chlorhexidine as per consultant preference and aseptically draped. The method of TIVAD insertion and closure was performed as per consultant preference. All cases used intra-operative fluoroscopy to confirm the position and a postoperative chest X-ray to assess for immediate complications as per hospital protocol.

Redcliffe Hospital protocol

All patients underwent a local MDT to determine ongoing appropriate management of their malignancy and need for long-term venous access. Informed written consent was gained prior to conducting the procedure. All procedures were performed as a day case under local anaesthetic by a radiologist and ultrasound guidance in the radiology department. Patients were prepped using alcoholic betadine or 2% chlorhexidine as per consultant preference and aseptically draped. TIVAD insertion was performed under standard protocol and closed as per consultant preference. Intra-operative fluoroscopy and postoperative chest X-ray were available. 

Inclusion criteria

Patients over the age of 18 who required the insertion of a TIVAD for chemotherapy were included. The TIVAD insertion techniques included were open cephalic vein cutdowns, percutaneous subclavian vein cannulation without the aid of ultrasound, and ultrasound-guided internal jugular vein cannulation. Patients requiring multiple insertions due to the removal of a TIVAD during their chemotherapy treatment were also included. All malignancies were included in this study.

Exclusion criteria

Patients under the age of 18 were excluded. Any TIVAD insertion for non-malignancy was excluded. Surgical manipulation of TIVADS without removing and re-inserting a new device was excluded.

Inclusion and exclusion criteria were entered into the ORMIS (Operating Room Management Information System) database generating a population list for the Caboolture and Redcliff Hospitals, which was used to collect the patient data. Patient files were reviewed on the electronic patient record to categorise demographics (age, sex, BMI, procedure indication), TIVAD insertion method, and complications. A detailed review of each patient’s electronic patient record was undertaken to determine any complications, removal of TIVADs, and any re-insertions.

Caboolture data were then organized into open cephalic cut-down, closed subclavian, and ultrasound-guided insertion. Redcliffe data were organized as ultrasound-guided insertion. Each procedure was scrutinized for intra-operative fluoroscopy and postoperative chest X-ray imaging. Complications were gathered by searching for emergency presentations, discharges, and further procedures related to their TIVAD. Descriptive data analysis was performed using the quantitative methodology only. Ethics approval was granted by the Royal Brisbane and Women’s Hospital human research ethics committee.

## Results

A total of 349 patients had undergone the insertion of a TIVAD at either Caboolture or Redcliffe Hospital during the study period. A total of 101 cases performed at Caboolture Hospital. 97 TIVADs (n=97) met the study inclusion criteria. Four patients were excluded from the Caboolture cohort: two external jugular vein approaches, one failure to advance the catheter despite multiple attempts resulting in case abortion, and one revision of the TIVAD subcutaneous position for patient comfort. A total of 248 patients underwent TIVAD insertion at Redcliffe Hospital (n=248). No patients were excluded from the Redcliffe Hospital cohort.

Table 1 outlines the Caboolture and Redcliff Hospital demographics. The mean age for TIVAD insertion at Caboolture Hospital was 63.7 years with a mean BMI of 29.1. Of the Caboolture group, 74 were female patients with a mean age of 63.7 years and 23 patients were male with a mean age of 66.8 years. The mean age in the radiology group was 61.6 years with a mean BMI of 28.9. Of the 248 patients, 198 were female with a mean age of 60.7 years and 50 were male with a mean age of 65.8 years.

**Table 1:** Patient demographics undergoing TIVAD insertion at Caboolture and Redcliffe Hospitals TIVAD: totally implantable venous access device

Demographics	Caboolture Hospital	Redcliffe Hospital
Total patients (n)	97	248
Mean BMI (kg)	29.1	28.9
Mean age	63.7	61.6
Total female (n)	74 (76.3%)	198 (79.8%)
Mean Female age	63.7	60.7
Total male (n)	23 (23.7%)	50 (20.2%)
Mean male age	66.8	65.8

The most common procedure performed by general surgeons was the percutaneous subclavian vein approach with 61 TIVAD insertions, followed by 29 ultrasound-guided internal jugular vein cannulations and seven open cephalic cut-downs. All 248 radiology inserted TIVADs were placed in the internal jugular vein under ultrasound guidance with 183 being right-sided and 65 left-sided. Within the radiology group (n=248), 247 TIVADs were successfully placed on the first attempt and one on the second attempt. Fifteen cases did not use intra-operative fluoroscopy or a postoperative chest X-ray. Only four cases received a postoperative chest X-ray; all of these also received intra-operative fluoroscopy. Table 2 shows the procedural breakdown of the surgical and radiology groups.

**Table 2:** Total number of TIVAD insertions by general surgeons at Caboolture Hospital and radiologists at Redcliffe Hospital based on anatomical location TIVAD: totally implantable venous access device

Caboolture TIVAD Insertion	Total (n=97)	Right	Left
Subclavian Vein	61 (62.3%)	48	13
Internal jugular vein	29 (30.0%)	27	2
Cephalic vein	7 (7.2%)	7	0
Redcliff TIVAD Insertion	Total (n=248)	Right	Left
Internal jugular vein	248 (100%)	183	65

Table 3 shows the overall surgical success rate of the first and second attempts for the TIVAD insertion techniques performed. Ultrasound-guided internal cannulation overall was the most successful, with 26 successful first attempts (89.7%) while inserting TIVADS. All three second internal jugular attempts were successful. Open cephalic cut-down followed with an 85.7% first-attempt success rate requiring only one second attempt, which was successful. Percutaneous subclavian cannulation had the lowest first-attempt success rate with 77.0% and a 14% second-attempt success rate. A total of four percutaneous subclavian cannulations were converted to USS-guided internal jugular insertions after two failed attempts.

**Table 3:** TIVAD insertion success rate of general surgeons at Caboolture Hospital based on the procedure and number of attempts required TIVAD: totally implantable venous access device

Surgical Technique	Total Inserted (n=97)	Successful First Attempt	Successful Second Attempt
Closed Subclavian Vein Cannulation	61	47 (77.0%)	14 (22.6%)
USS-Guided Internal Jugular Vein Cannulation	29	26 (89.7%)	3 (33.3%)
Open Cephalic Vein Cut-Down	7	6 (85.7%)	1 (14.3%)

Overall, there was a 16.4% (16/97 cases) rate of surgical complications. Complications included five pneumothorax, five port malfunctions, two subcutaneous haematomas, two infections, one thrombus, and one right atrial injury. Percutaneous subclavian cannulation had the highest rate of complications with four life-threatening complications, including three cases of pneumothorax and one right atrial injury requiring transfer to a tertiary referral centre for cardiothoracic support. Port malfunction included three migrations, and two instances of inability to access the port. Details of all surgical complications are outlined by procedure type in Table 4.

**Table 4:** Surgical complications based on TIVAD insertion and type TIVAD: totally implantable venous access device

Surgical Complication	Total	Subclavian Vein	Cephalic Vein	Internal Jugular
Pneumothorax	5	3	0	2
Port Malfunction	5	2	2	1
Migration	3	2	0	1
Inability to access	2	0	2	0
Subcutaneous Haematoma	2	1	1	0
Infection	2	1	0	1
Thrombus	1	1	0	0
Right Atrial Injury	1	1	0	0

There was a 15.3% (38/248 cases) rate of complications from insertion of TIVADs by radiologists. Complications included 22 port-related infections, 14 port malfunctions, one procedural haemorrhage controlled with pressure, and one right atrial injury requiring transfer to a cardiothoracic unit. One of the cases resulted in sepsis within 24 hours requiring surgical washout and replacement in theatre. Details of the complications are listed in Table 5.

**Table 5:** Radiology TIVAD insertion complications Total of 38 complications from 248 ultrasound-guided internal jugular vein insertions TIVAD: totally implantable venous access device

Radiology insertion complications	Total (n=248)
Infection	22 (8.9%)
Cellulitis	13 (5.2%)
Port bacteraemia	5 (2.0%)
Port abscess	2 (0.81%)
Incision dehiscence	2 (0.81%)
Port malfunction	14 (5.6%)
Unable to access	6 (2.4%)
Port flipping	5 (2.0%)
Splitting	1 (0.4%)
Kinking	1 (0.4%)
Migration	1 (0.4%)
Haemorrhage	1 (0.4%)
Right atrial injury	1 (0.4%)

## Discussion

The number of patients with cancer is increasing, with a subsequent rise in the prescription of chemotherapeutic regimens and, hence, the need for TIVADS is growing [1,5]. Presently there are no clear guidelines for TIVAD insertions, and this has resulted in the development of multiple techniques for insertion [2-3]. General surgeons are required to maintain the technical skills to perform these procedures, as they are often required to perform TIVAD insertions. Additionally, with the growing number of cancers diagnosed the medical community is beginning to see more radiologists performing ultrasound-guided TIVAD insertions with great effect [6,12]. Overall, these procedures have been shown to be effective and safe but do have associated risks [1-3,5-6]. Complications include infection, thrombosis, port malfunctions but occasionally may include life-threatening pneumothorax, haemothorax, mediastinal injuries, and great vessel injury [6].

Percutaneous subclavian cannulation is commonly performed by both surgeons and non-surgical practitioners due to its ease and high success rate [13-14]. Interestingly, despite its procedural simplicity percutaneous subclavian cannulation often requires multiple puncture attempts and is associated with increased rates of great vessel and lung injuries compared to other techniques [5,14]. Knebel et al. recently demonstrated in the PORTAS-3 trial that the cephalic cut-down had a statistically significant reduction in the rate of pneumothorax and haemothorax compared to percutaneous subclavian cannulation without an ultrasound [5]. One drawback of the open cut-down is the anatomical variation in location and size of the cephalic vein, resulting in a higher fail rate and conversion to another insertion technique [2,6]. However, in 2009, Knebel et al. demonstrated that the open cephalic cut-down had success rates of >90% when employing the rescue mechanism compared to the percutaneous subclavian cannulation without ultrasound [7].

This study is the first to our knowledge that directly compares the success rates of general surgeons and radiologists while using any TIVAD insertion technique. Radiologists had an overall 99.6% first-attempt success rate while performing ultrasound-guided internal jugular cannulations compared to the surgical groups' overall first-attempt success rate of 81.4%. Percutaneous subclavian cannulation was the most common procedure performed by the surgeons with the lowest first-attempt success rate of 77.0% (47/69 cases), which has been regularly reported as a downfall in its utility [5,14]. Similar to the radiologists, the general surgeons showed a high first-attempt success rate of 89.7% while performing ultrasound-guided cannulation, as it was the second-most common procedure. Eighty-five point seven per cent (85.7%; 6/7 cases) open cephalic venous cut-downs were successful on the first attempt while using the rescue technique. Unfortunately, it is difficult to accurately compare this procedure to other approaches due to such few attempts. One explanation for the near-perfect success rate of the radiology group is that radiologists attempt internal jugular cannulation as a general practice and perform the procedure frequently. There was great variability in the preferences and procedural attempts amongst the surgeons, making direct comparison difficult to interpret.

The authors of this study considered 9.3% of the surgical group complications to be potentially life-threatening, which included five pneumothoraxes, three subclavian vessel injuries resulting in haematomas, and one right atrial injury requiring transfer. Two pneumothoraxes occurred in internal jugular TIVADs after failed percutaneous subclavian cannulations, making it difficult to ascertain the cause of the injury, as the complication was picked up on a routine chest X-ray. The pneumothorax complications seen in the percutaneous subclavian group are consistent with the results in the recent PORTAS-3 trial where percutaneous subclavian cannulation without ultrasound guidance showed significantly higher rates of pneumothorax or haemothorax compared to open cephalic cut-down [5]. Furthermore, this study’s surgical pneumothorax complication rate of 5.2% is comparable to the 4% reported in the literature [5]. The radiologists had no cases of pneumothorax reported, and we suspect this is largely explained by the anatomical distance of the internal jugular vein from the pleura as well as the procedure being guided by ultrasound. The radiology cohort had a single life-threatening complication with a right atrial injury requiring observations.

Overall, the radiology group had a higher rate and variety of postoperative infections compared to the surgical group. Importantly, 33% (7/22 infections) of the radiologists’ postoperative infections required replacement, whereas the surgeons only had two postoperative infections, both requiring replacement. One radiological case developed postoperative sepsis within 24 hours, requiring immediate take-back for washout and TIVAD replacement in the theatre. The difference in infection rates may be explained by the limitations of sterility and aseptic technique within the radiology department, whereas all surgical cases were performed in the operating theatre. Another explanation between the infection rate of the two groups may also be the surgeons’ superior suturing technique and tissue closure compared to their radiology colleagues.

There are several limitations to this study. Firstly, the authors of this study acknowledge that many complications may have been missed by patients who presented to their general practitioner for minor complications, including wound management. Secondly, inferential statistics were unable to be generated due to the large number of variables between the types of TIVAD insertions as well as the skewed number of cases in each group. Third, the pneumothorax complications may be underreported, as most radiology cases did not perform postoperative chest X-rays and small pneumothoraces may have been missed. Larger study groups will be required to help generate statistical significance between the surgical interventions as well as both research arms.

## Conclusions

This retrospective review demonstrates that ultrasound-guided cannulation of the internal jugular vein had the highest number of successful first attempts while inserting TIVADs in both the surgical and radiological groups. This review also suggests that TIVAD insertion can be reliably inserted by both general surgeons and radiologists provided that all attempts are made to maximise sterility. This study does not provide enough data to suggest a procedural guideline for insertion, but the authors recognize the role of cephalic and subclavian insertions, which should be discussed on a case-by-case basis in association with patients’ preferences.
